# Multi-Omics Analysis Reveals MicroRNAs Associated With Cardiometabolic Traits

**DOI:** 10.3389/fgene.2020.00110

**Published:** 2020-02-27

**Authors:** Michelle M. J. Mens, Silvana C. E. Maas, Jaco Klap, Gerrit Jan Weverling, Paul Klatser, Just P. J. Brakenhoff, Joyce B. J. van Meurs, André G. Uitterlinden, M. Arfan Ikram, Maryam Kavousi, Mohsen Ghanbari

**Affiliations:** ^1^ Department of Epidemiology, Erasmus MC University Medical Center Rotterdam, Rotterdam, Netherlands; ^2^ Department of Genetic Identification, Erasmus MC University Medical Center Rotterdam, Rotterdam, Netherlands; ^3^ World Without Disease Accelerator, Data Sciences & Prevention Biomarkers, Johnson & Johnson, Leiden, Netherlands; ^4^ Department of Internal Medicine, Erasmus MC University Medical Center Rotterdam, Rotterdam, Netherlands

**Keywords:** cardiometabolic traits, microRNA, multi-omics data, GWAS, EWAS

## Abstract

MicroRNAs (miRNAs) are non-coding RNA molecules that regulate gene expression. Extensive research has explored the role of miRNAs in the risk for type 2 diabetes (T2D) and coronary heart disease (CHD) using single-omics data, but much less by leveraging population-based omics data. Here we aimed to conduct a multi-omics analysis to identify miRNAs associated with cardiometabolic risk factors and diseases. First, we used publicly available summary statistics from large-scale genome-wide association studies to find genetic variants in miRNA-related sequences associated with various cardiometabolic traits, including lipid and obesity-related traits, glycemic indices, blood pressure, and disease prevalence of T2D and CHD. Then, we used DNA methylation and miRNA expression data from participants of the Rotterdam Study to further investigate the link between associated miRNAs and cardiometabolic traits. After correcting for multiple testing, 180 genetic variants annotated to 67 independent miRNAs were associated with the studied traits. Alterations in DNA methylation levels of CpG sites annotated to 38 of these miRNAs were associated with the same trait(s). Moreover, we found that plasma expression levels of 8 of the 67 identified miRNAs were also associated with the same trait. Integrating the results of different omics data showed miR-10b-5p, miR-148a-3p, miR-125b-5p, and miR-100-5p to be strongly linked to lipid traits. Collectively, our multi-omics analysis revealed multiple miRNAs that could be considered as potential biomarkers for early diagnosis and progression of cardiometabolic diseases.

## Introduction

Type 2 diabetes mellitus (T2D) is a complex metabolic disease that is characterized by insulin resistance and impairment of insulin secretion, which leads to hyperglycemia. The presence of T2D leads to a two- to four-fold increase risk of developing coronary heart disease (CHD) (Kannel and Mcgee, 1979), which is among the leading causes of morbidity and mortality worldwide (Roth et al., 2018). Many risk factors are identified as mediators of these diseases, including hypertension, dyslipidemia, central adiposity and elevated blood glucose, which are together known as cardiometabolic traits (Wilson et al., 2005). Despite substantial advances in diagnosis and widely prescribed drugs for these diseases, their rate continue to increase worldwide, emphasizing the need for deeper insights into underlying mechanisms and innovative therapeutic strategies. Cardiometabolic traits and diseases have underlying genetic components and many loci have been discovered through large-scale genome- and epigenome-wide association studies (Peloso et al., 2014; De Rosa et al., 2018). However, most of the identified genetic variants do not affect protein sequences, but are thought to affect gene regulation. One of the potential regulatory mechanisms involved might be microRNAs (miRNAs).

MiRNAs represent a class of small non-coding RNAs, which function as post-transcriptional regulators of gene expression *via* targeting the 3’ untranslated region of target transcripts (Friedman et al., 2009). Over the past years, miRNAs have emerged as key regulators of biological processes underlying T2D and CHD. In this context, aberrant expression and function of miRNAs, such as miR-33, miR-208, miR-133, and miR-124, have been shown to be associated with lipid metabolism, insulin secretion, myocardial infarction and T2D (Stauffer et al., 2013; Rotllan et al., 2016; Wang et al., 2017). Most of the disease-associated miRNAs have been discovered in cells originated from tissue of interest in small number of samples or animal studies. But advances in high-throughput technologies make it possible to study miRNAs in a population-based manner. In particular cell-derived vesicles, known as exosomes, release miRNAs in the blood stream that are very stable and can be used as biomarkers for disease (Grasedieck et al., 2012).

Similar to other regulatory RNA molecules, the function and expression of miRNAs can be affected by genetic variants. Single-nucleotide polymorphisms (SNPs) can occur at various stages of the miRNA biogenesis including precursor- and mature miRNA sequences (Gong et al., 2012) as well as within regulatory elements, such as promoter regions (de Rie et al., 2017). Also, DNA methylation can control transcription, which have been reported to be associated with the expression level of miRNAs (Huan et al., 2019). In this context, epigenome-wide association studies (EWAS) have shown that altered DNA methylation within miRNA promoters is associated with miRNAs expression levels and therewith modify disease risk (Aure et al., 2013). However, previous studies are mainly based on single omics data or small sample size (de Candia et al., 2017; Hijmans et al., 2018). As each type of omics data provides associations that can be useful for detecting development or progression of disease, integrating different omics layers can limit passive correlations and provide a more comprehensive view of the disease biology.

In this study, we applied a multi-omics approach to identify miRNAs associated with cardiometabolic traits. First, we identified genetic variants in miRNA sequences and their potential regulatory regions associated with different cardiometabolic risk factors and diseases using genetic association data from the available genome-wide association studies (GWAS). We then integrated population-based DNA methylation and miRNA expression data from the Rotterdam Study to link omics layers, strengthening the association of the identified miRNAs with cardiometabolic traits. We envision that the identified miRNAs could be considered as potential biomarkers for early diagnosis of cardiometabolic diseases.

## Material and Methods

**Figure 1:**
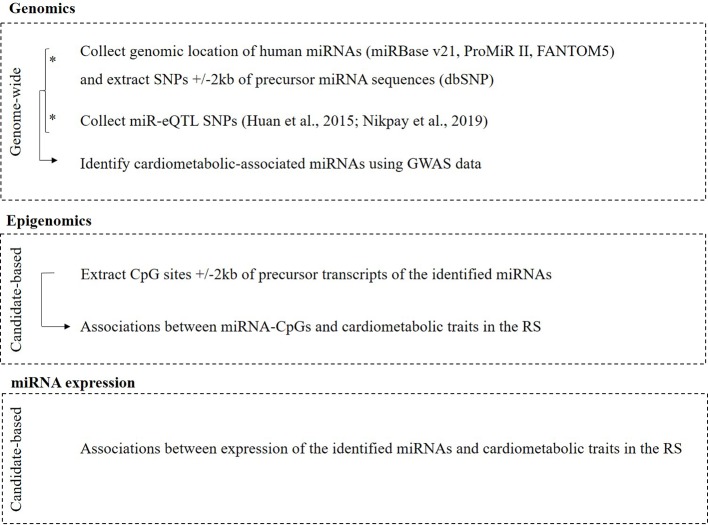
Overview of the multi-omics layers used in this study.

A graphical overview of the multi-omics approach used in this study is illustrated in **Figure 1**.

### Retrieval of SNPs in miRNA-Related Regions

The primary transcripts of miRNAs for the processing to mature miRNAs are approximately 3–4kb in length (Saini et al., 2007). We collected the genomic position of all human miRNAs employing the miRBase database (v21) (Kozomara and Griffiths-Jones, 2014), ProMiR II (Nam et al., 2006), and FANTOM5 (de Rie et al., 2017). Using dbSNP database (Sherry et al., 2001), we extracted 18,545 SNPs located in +/-2kb of the precursor miRNA sequences (pre-miRNA) of 1,554 known miRNAs. Of these, 2,420 SNPs are located in pre- and mature sequences of miRNAs. Genetic variants have been found to alter miRNA expression and are known as miRNA expression quantitative trait loci (miR-eQTLs). To this end, we included 5,528 miR-eQTLs that change the expression of 221 mature miRNA using data from the Framingham Heart Study (FHS) (Huan et al., 2015) and from the Ottawa Hospital Bariatric Centre (Nikpay et al., 2019). The FHS focused on *cis*-miR-eQTLs, of which the majority was located 300–500kb away from their target miRNA. Nikpay et al. (2019) investigated both *cis*-miR-eQTLs and *trans*-miR-eQTLs, however, they reported similar to the FHS that most cis-miR-eQTLs were distal regulators of the miRNAs. There were 83 miR-eQTLs overlapping with the SNPs in +/-2kb of the precursor miRNA sequence. Altogether, 23,990 unique SNPs were included in our analysis.

The genomic location of miRNAs can be discriminated among intergenic and intragenic. Roughly half of the known miRNAs are found to be transcribed from intergenic regions of the genome, suggesting that these miRNAs are transcribed under independent control of regulatory elements (Liu et al., 2018). The intragenic miRNAs are embedded within sequences of protein-coding genes, including intronic and exonic regions. If the intragenic miRNA and its host gene share the same promoter, the miRNA is likely to be co-expressed with the host gene (Lutter et al., 2010). Here, the genomic location of the identified miRNAs was obtained using miRIAD (Hinske et al., 2014).

### Genome-Wide Association Studies of Cardiometabolic Traits

Cardiometabolic risk factors and diseases in this study were classified into four specific trait groups based on their shared pathophysiology and underlying pathways. These include (i) Anthropometric traits: body mass index (BMI), waist to hip ratio (WHR) and waist circumference (WC); (ii) Glycemic traits: fasting glucose (FG), glucose 2 hours (G2H), fasting insulin (FI), proinsulin (Pro-Ins), hemoglobin A1c (HbA1c), homeostatic model assessment of insulin resistance (HOMA-IR), β-cell function (HOMA-β), and type 2 diabetes mellitus (T2D); (iii) Lipid traits: low-density lipoprotein (LDL), high-density lipoprotein (HDL), total serum cholesterol (TC), and triglycerides (TG); and (iv) Cardiovascular traits: coronary artery disease (CAD), diastolic (DBP), and systolic blood pressure (SBP). To test the association of miRNA-related SNPs with cardiometabolic traits, we used publicly available GWAS summary statistics. A description of GWAS meta-analysis data and corresponding consortia used in this study is provided in **Supplementary Table S1**. To obtain the number of independent SNPs, we used the linkage disequilibrium (LD) based SNP pruning in PLINK (http://pngu.mgh.harvard.edu/~purcell/plink/), in which we excluded the SNPs with R^2^ > 0.7. Bonferroni correction was used to adjust for multiple testing based on the number of independent SNPs available in the GWAS data (HapMap or 1000G project imputed data).

### Prioritization of miRNA-Related SNPs Associated With Cardiometabolic Traits

For miRNA-related SNPs significantly associated with cardiometabolic traits, we performed *in silico* analysis to prioritize the SNPs that are more likely to be functional in their corresponding loci based on the following criteria: (i) association between the miRNA-related SNP and the cardiometabolic trait, (ii) association between the miRNA-related SNP and the expression level of miRNA/miRNA hosting gene, and (iii) expression of the miRNA in tissues relevant to cardiometabolic traits. In this regard, regional association plots were generated (using LocusZoom web tool, Version 1.1) to visualize the physical position and evaluate the association of the cardiometabolic traits with the miRNA-related SNP and its proxy SNPs (R^2^ > 0.8) in the corresponding locus: (i) To explore whether the SNP is associated with the expression of related miRNA or miRNA hosting genes in relevant tissues (e.g., adipose tissue, liver, pancreas, muscle and blood), we used eQTL data from GTEx Portal (https://www.gtexportal.org/home/), (ii) we used two online databases; miRmine and Human miRNA tissue atlas (Ludwig et al., 2016; Panwar et al., 2017) to test where a miRNA is expressed in tissues relevant to cardiometabolic traits (e.g., adipose tissue, liver, pancreas, muscle, and blood), (iii) the Vienna RNAfold algorithm was used to check miRNA secondary structure and free energy changes with wild-type and mutant alleles of SNPs located in miRNA sequences (Lorenz et al., 2011).

### Determination of Methylation Quantitative Trait Loci (me-QTLs)

To determine if the identified SNPs have an effect on the methylation levels of CpG sites (me-QTLs), we used data of a recent me-QTL study performed in five cohorts, including the RS, with a total of 3,841 individuals (Bonder et al., 2017). We incorporated both *cis*-me-QTLs and *trans*-me-QTLs. Where *cis*-me-QTLs were defined as the effect of SNPs on the methylation levels of a CpG sites no further than 250kb apart, *trans*-me-QTLs were defined as the effect of distal SNPs on the CpG methylation levels. Details on the me-QTL mapping are described elsewhere (Bonder et al., 2017). We tested if the cardiometabolic-associated SNPs found in the current study were identified as me-QTLs.

### DNA Methylation Analysis in the Rotterdam Study

The Rotterdam Study (RS) is a large prospective population-based cohort study conducted among middle-aged and elderly people in the suburb Ommoord in Rotterdam, the Netherlands. In 1989, 7,983 inhabitants aged 55 and older were recruited in the first cohort (RS-I) (78% of 10,215 invitees). In 2000, the RS was extended with a second cohort of 3,011 participants that moved to Ommoord or turned 55 years old (RS-II). In 2006, the third cohort (RS-III) was initiated in which inhabitants aged 45-54 years were invited and included 3,932 participants. A detailed description of RS can be found elsewhere (Ikram et al., 2017). In the current study, we used DNA methylation data from a random subset (n = 717) of the third visit of RS-II (RS-II-3) and second visit of RS-III (RS-III-2) and a random subset (n = 721) of the first visit of RS-III (RS-III-1). There was no overlap in participants. The RS has been approved by the institutional review board (Medical Ethics Committee) of the Erasmus Medical Center and by the review board of The Netherlands Ministry of Health, Welfare and Sports. All participants gave written consent before participation in the study. Participant characteristics are presented in **Supplementary Table S2**.

DNA was extracted from whole peripheral blood using standardized salting out methods, of which 500ng was bisulfite treated using the Zymo EZ-96 DNA methylation kit (Zymo Research, Irvine, CA, USA). Bisulfite converted DNA was hybridized to the Illumina Human 450K array (Illumina, San Diego, CA, USA), according to manufacturer’s protocol. Data preprocessing was performed using an R programming pipeline based on the pipeline developed by Touleimat and Tost (Touleimat and Tost, 2012). The genome coordinates provided by Illumina (GRCh37/hg19) were used to identify independent loci. We extracted 12,939 unique CpGs located in +/-2kb of the pre-miRNA sequences using the Illumina450K array annotation file as provided by Illumina (Sandoval et al., 2011). Among these, 12,617 CpGs were located in the regulatory region of 1,269 miRNAs and 450 CpGs were located in the pre- and mature sequence of 391 miRNAs. We tested the association of these CpGs with different cardiometabolic traits using linear mixed models. Data collection on these traits in the RS is described in **Supplementary Methods**. The models were adjusted for age, gender, current smoking, blood cell counts (monocytes, granulocytes, lymphocytes) as fixed effects and technical covariates as random effects. Models were further adjusted for covariates per group as follows: (i) for Anthropometric traits we adjusted WC and WHR for BMI, (ii) for Glycemic traits we adjusted for BMI and diabetic medication, (iii) for Lipid traits we adjusted for BMI and lipid medication, and (iv) for Cardiovascular traits we adjusted for BMI, blood pressure lowering medication and lipid medication. A candidate-based approach was used to sought overlap between identified miRNAs. A nominal p-value of <0.05 was found to be significant.

### Determination of miR-eQTMs

To identify association between the methylation level of CpGs and the expression of miRNAs (miR-eQTMs), we used miR-eQTM data from a recent study (Huan et al., 2019). The latter study analyzed associations of expression levels of 283 miRNAs with methylation of CpGs from 3,565 individuals, in which they identified 227 miR-eQTMs at FDR < 0.01. We tested if any of the cardiometabolic-associated CpGs in the current study was among the identified miR-eQTM (Huan et al., 2019).

### MiRNA Expression Profiling in the Rotterdam Study

We performed miRNA expression analysis in 2,000 RS participants, including a random subset (n = 1,000) of the fourth visit of RS-I (RS-I-4) and a random subset (n = 1,000) of the second visit of RS-II (RS-II-2). Plasma miRNA levels were determined using the HTG EdgeSeq miRNA Whole Transcriptome Assay (WTA), which measures the expression of 2,083 mature human miRNAs (HTG Molecular Diagnostics, Tuscon, AZ, USA) and using the Illumina NextSeq 500 sequencer (Illumina, San Diego, CA, USA). The WTA characterizes miRNA expression patterns, and measures the expression of 13 housekeeping genes, that allows flexibility in data normalization and analysis. Quantification of miRNA expression was based on counts per million (CPM). Log2 transformation of CPM was used as standardization and adjustment for total reads within each sample. MiRNAs with Log2 CPM < 1.0 were indicated as not expressed in the samples. The lower limit of quantification (LLOQ) was used to select well-expressed miRNAs. The LLOQ level was based on a monotonic decreasing spline curve fit between the means and standard deviations of all miRNAs. In our definition well-expressed miRNA levels in plasma were those with >50% values above LLOQ. Out of the 2,083 measured miRNAs, 591 miRNAs were expressed at good levels in plasma.

The miRNAs significantly associated with cardiometabolic traits, in the genetic association studies, were tested for the association of their plasma expression levels with the same cardiometabolic trait(s). Linear models were used to test the association between available continuous traits in the RS (incl. BMI, WC, WHR, FG, HDL, TC, SBD, and DBP) and miRNA expression. Additionally, we used binomial models to test the association between disease prevalence (incl. T2D and CHD) and miRNA expression. We used the cardiometabolic traits as dependent variable and plasma miRNAs level as explanatory variable, adjusting for age, gender and current smoking. Models were further adjusted for covariates per group as follows: (i) for Anthropometric traits we adjusted WC and WHR for BMI, (ii) for Glycemic traits we adjusted for BMI and diabetic medication, (iii) for Lipid traits we adjusted for BMI and lipid medication, and (iv) for Cardiovascular traits we adjusted for BMI, blood pressure lowering medication and lipid medication. A candidate-based approach was used to sought overlap between identified miRNAs. A nominal p-value of <0.05 was found to be significant.

In addition, we extracted strongly validated target genes, defined as being validated by western blot and/or luciferase reporter assay, of the identified miRNAs from the miRTarBase database (Chou et al., 2018). Next, we extracted SNPs in these target genes and tested their associations with cardiometabolic traits using summary statistics of previously mentioned GWAS data.

## Results

### Association of miRNA-SNPs With Cardiometabolic Traits and Diseases

**Figure 2:**
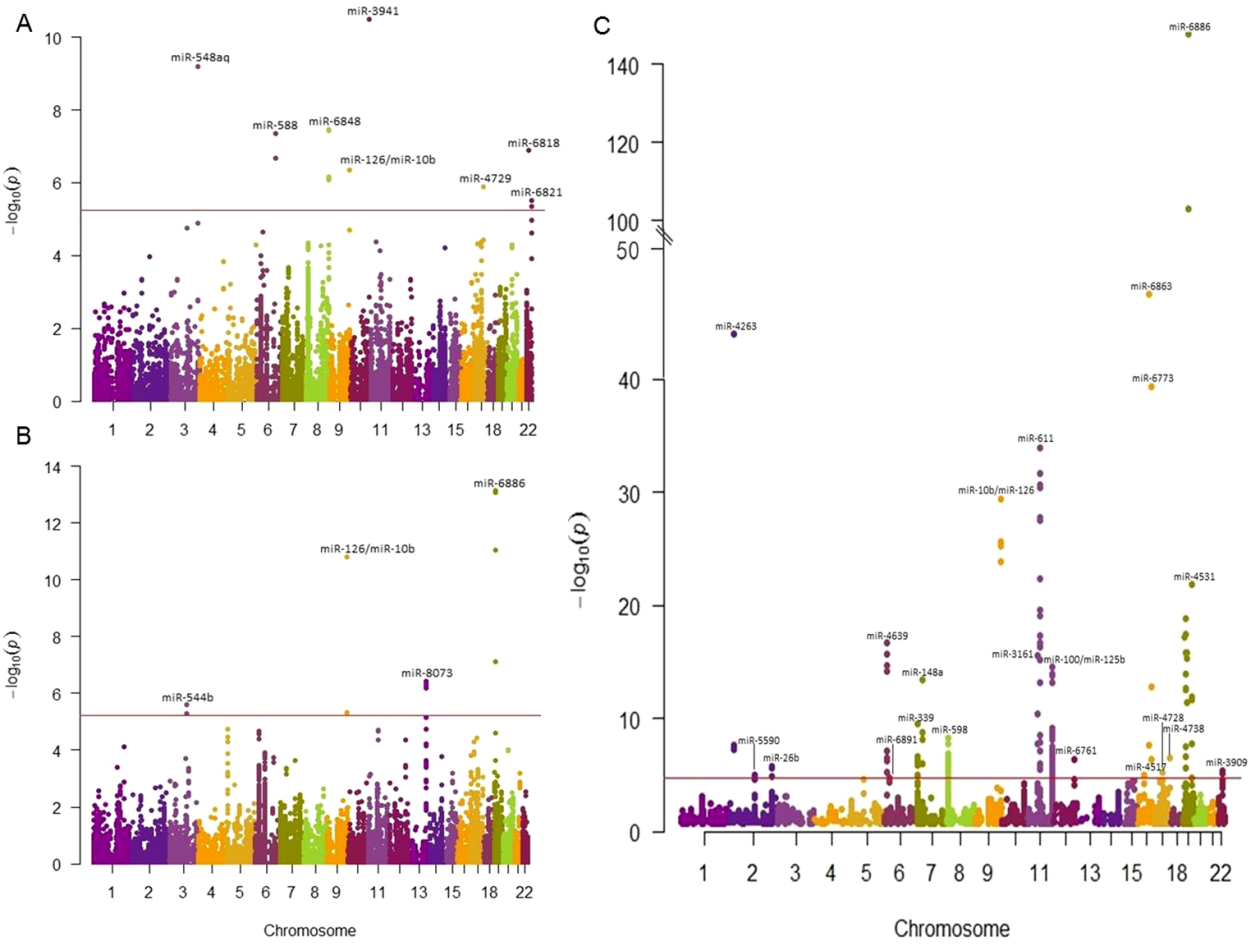
Manhattan plots showing the association of miRNA-SNPs with T2D, CAD, and lipid traits. The association miRNA-related SNPs and cardiometabolic traits were examined using the publicly available GWAS data. We reported the most significantly associated miRNA of each SNP loci. The horizontal red line indicates the study significance threshold. **(A)** Manhattan plot showing the association of miRNA-SNPs with T2D in which 12 SNPs in 8 miRNAs passed the significant threshold. **(B)** Manhattan plot showing the association of miRNA-SNPs with CAD in which 13 SNPs in 9 miRNAs passed the significance threshold. **(C)** Manhattan plot showing the association of miRNA-SNPs with lipid traits in which 107 SNPs in 36 miRNAs passed the significant threshold. When SNPs were present in more traits, the most associated SNP was plotted.

Out of 23,990 miRNA-related SNPs, 2,358 independent SNPs were present in the GWAS data based on HapMap and 8,652 independent SNPs were present in the 1000G project. Bonferroni correction was used to set the significance threshold, at p-value <2.12×10^-5^ (0.05/2,358) for GWAS with HapMap imputed data and p-value < 5.78×10^-6^ (0.05/8,652) for GWAS with 1000 Genomes project imputed data. Of these, 180 SNPs annotated to 67 miRNAs passed the significance threshold to be associated with at least one cardiometabolic trait (**Table 1**). Out of the 180 identified SNPs, 89 SNPs were located in +/-2kb of 57 primary miRNA transcripts (**Supplementary Table S3**) and 92 SNPs were among the previously reported miR-eQTLs of 15 mature miRNAs (**Supplementary Table S4**). Manhattan plots illustrated in **Figure 2** present the miRNA-annotated genetic variants associated with lipid traits and the prevalence of T2D and CHD. **Table 2** shows the top miRNA-related SNPs associated with cardiometabolic traits, which were annotated to 20 miRNAs.

**Table 1:** Description of genome-wide association studies (GWAS) of cardiometabolic traits and associated miRNA single-nucleotide polymorphisms (SNPs).

Phenotype	Consortium	SNPs in +/-2kb miR^*^	SNPs in miR-seq^*^	SNPs in miR-QTL^*^	Associated miR loci^†^
**Anthropometric traits**					
Body-mass index	GIANT (Locke et al., 2015)	9	0	9	7
Waist to hip ratio	GIANT (Shungin et al., 2015)	2	1	1	4
Waist circumference	GIANT (Shungin et al., 2015)	10	0	1	8
**Glycemic traits**					
Glucose fasting	MAGIC (Manning et al., 2012)	3	0	1	4
Glucose after 2h	MAGIC (Saxena et al., 2010)	0	0	0	0
Insulin fasting	MAGIC (Manning et al., 2012)	1	0	2	2
Proinsulin	MAGIC(Strawbridge et al., 2011)	3	0	4	3
HbA1c	MAGIC (Wheeler et al., 2017)	1	0	15	4
HOMA-IR	MAGIC (Dupuis et al., 2010)	0	0	0	0
HOMA-β	MAGIC (Dupuis et al., 2010)	0	0	0	0
Type 2 diabetes	DIAGRAM (Scott et al., 2017)	12	0	1	8
**Lipid traits**					
LDL	GLGC (Willer et al., 2013)	22	1	20	11
HDL	GLGC (Willer et al., 2013)	12	1	23	9
Total cholesterol	GLGC (Willer et al., 2013)	26	1	40	13
Triglyceride	GLGC (Willer et al., 2013)	8	1	27	7
**Cardiovascular traits**					
CAD	CARDIoGRM plusC4D (Nikpay et al., 2015)	10	0	2	4
DBP	ICBP (International Consortium for Blood Pressure Genome-Wide Association et al., 2011)	3	0	–	2
SBP	ICBP (International Consortium for Blood Pressure Genome-Wide Association et al., 2011)	2	0	–	2

Shown are SNPs located within +/-2kb of primary miRNA transcripts, pre- and mature miRNA sequences, miRNA-eQTL SNPs.

*Number of SNPs that passed the significance threshold (p-value < 2.12x10^-5^ for SNPs imputed with HapMap and p-value < 5.78x10^-6^ for SNPs imputed with 1000G).

^†^Number of independent loci.

**Table 2:** The top 20 miRNAs with single-nucleotide polymorphisms (SNPs) in related regions association with cardiometabolic traits.

miRNA	SNPID	Chr.	Position	Alleles (A/R)	Annotated gene	Genomic location miRNA	Associated trait	β-coefficient	P value
**miR-6886^†^**	rs17248720	19	11198187	C/T	*LDLR*	Intronic	LDL	0.226	2.40x10^-148^
**miR-6863^†^**	rs13306673	16	56900931	C/T	*SLC12A3*	Intronic	HDL	0.098	2.76x10^-48^
**miR-4263^†^**	rs2305929	2	28113911	G/A	*BRE*	Intronic	TG	0.064	1.13x10^-44^
**miR-6773^†^**	rs8057119	16	68268836	T/C	*ESRP2*	Intronic	HDL	0.072	5.21x10^-40^
**miR-611^†^**	rs174538	11	61560081	G/A	*THEM258*	Exonic	LDL	0.050	1.07x10^-34^
**miR-1908-5p^‡^**	rs174548	11	61571348	C/G	*FADS1*	Exonic	LDL	0.047	2.29x10^-31^
**miR-10b-5p/126-5p^‡^**	rs532436	9	136149830	A/G	*ABO*	Exonic/Intronic	LDL	0.079	4.02x10^-30^
**miR-4721^†^**	rs4788099	16	28763228	G/A	*TUMF*	Exonic	BMI	0.031	1.09x10^-24^
**miR-4531^†^**	rs6509170	19	45159636	C/A	*LOC107985305*	Intronic	LDL	0.127	1.54x10^-22^
**miR-199a-1^†^**	rs11085748	19	10927540	T/C	*DNM2*	Intronic	LDL	0.055	1.46x10^-19^
**miR-4999^†^**	rs7254882	19	8359822	C/T	*MIR4999*	Intergenic	HDL	0.033	6.66x10^-18^
**miR-4639^†^**	rs3757354	6	16127407	C/T	*MYLIP*	Intronic	LDL	0.038	2.09x10^-17^
**miR-640^†^**	rs1000237	19	19518316	T/A	*GATAD2A*	Intronic	TG	0.033	1.61x10^-16^
**miR-3161^†^**	rs79837139	11	48000780	C/T	*PTPRJ*	Intronic	HDL	0.062	2.99x10^-16^
**miR-100-5p/125b-5p^‡^**	rs7117842	11	122663796	C/T	*UBASH3B*	Intergenic/Intergenic	TC	0.029	2.48x10^-15^
**miR-148a^†^**	rs4722551	7	25991826	C/T	*MIR148A*	Intergenic	LDL	0.039	3.95x10^-14^
**miR-139^†^**	rs11605042	11	72700619	A/G	*ARAP1*	Intronic	Pro-Ins	-0.053	5.24x10^-13^
**miR-3941^†^**	rs71486610	10	124134803	C/G	*PLEKHA1*	Intronic	T2D	-0.081	3.30x10^-11^
**miR-6745^†^**	rs901750	11	47209472	A/G	*PACSIN3*	Intronic	HDL	0.024	3.95x10^-11^
miR-196a-2-3p^*^	rs11614913	12	53991815	C/T	*MIR196A2*	Intergenic	WHR	0.029	6.90x10^-11^

^*^SNP located in pre- and mature miRNA sequence.

^†^SNP located within +/- 2kb of primary miRNA transcript.

^‡^miR-eQTL SNP.

**Figure 3:**
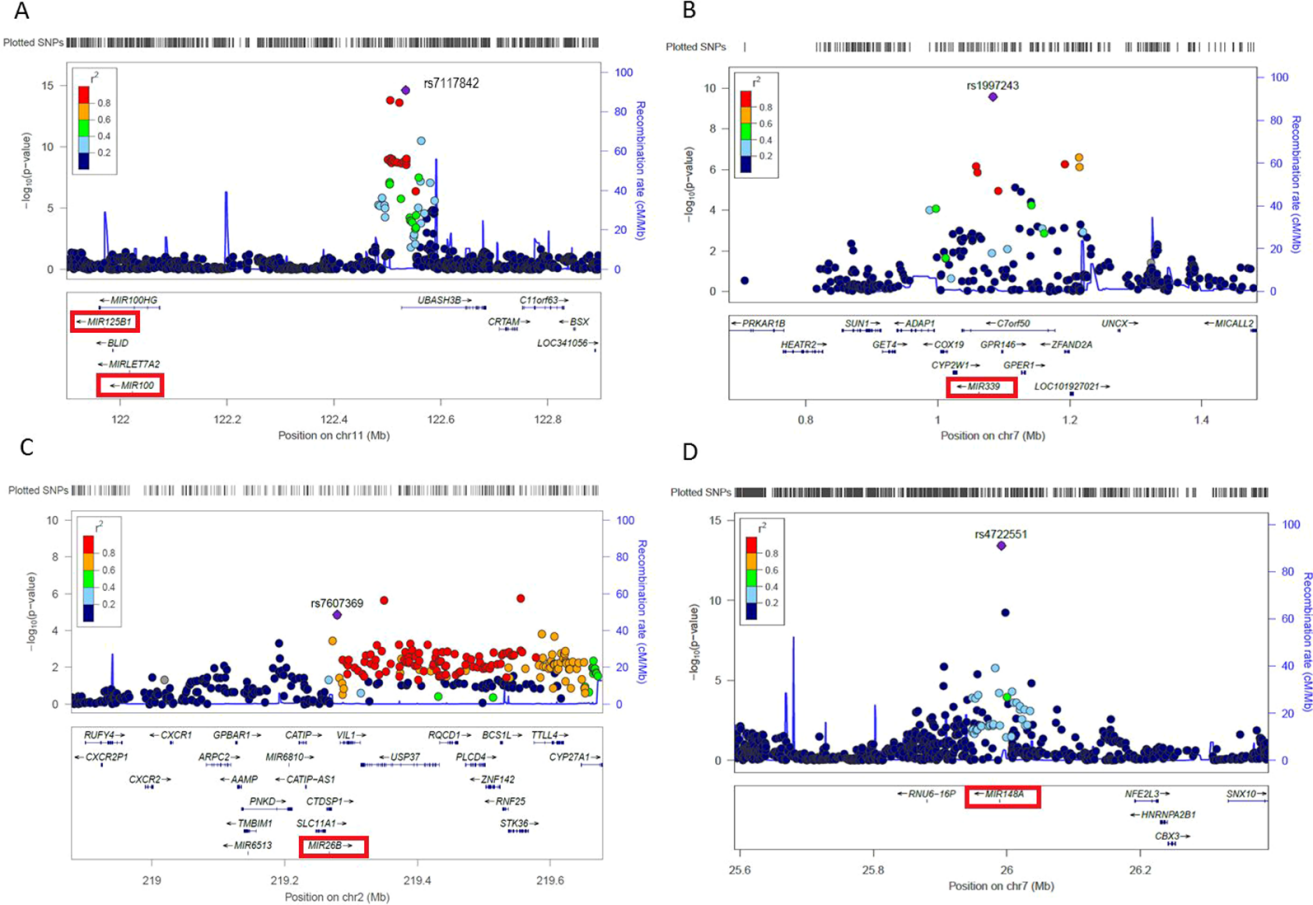
Regional plots showing the association of four top miRNA-SNPs with cardiometabolic traits. The most significant SNP in the region, according to P-value, is represented by a purple diamond, and the degree of linkage disequilibrium of other SNPs in the region to the lead SNP is representative by the color scale shown in the legend. Genes are illustrated below. The associated miRNA is illustrated with a red box. **(A)** Regional plot showing the association of rs7117842 located ~512kb upstream of miR-100-5p/125b-5p with TC, LDL and HDL on the Chr11q24.1 locus. **(B)** Regional plot showing the association of rs1997243 located ~21kb upstream of the primary transcript of miR-339-3p with TC and HDL on the Chr7p22.3 locus. **(C)** Regional plot showing the association of rs7607369 located ~11.7kb upstream of the primary transcript of miR-26b-5p with BMI and TG on the Chr2q35 locus. **(D)** Regional plot showing the association of rs472551 located ~2kb upstream of the primary transcript of miR-148a with LDL, TG, and TC on the Chr7p15.2 locus.

In order to prioritize miRNA-related SNPs based on potential functionality in relation to the associated cardiometabolic traits, we created regional association plots to visualize the LD of miRNA SNP with the top SNP in the corresponding locus (**Figure 3**). We found three top SNPs in their loci, including rs7117842 associated with TC (p = 2.48×10^-15^, β = v0.029) and located ~512kb upstream of miR-100-5p/miR-125b-5p (**Figure 3A**), rs1997243 associated with TC (p = 2.72×10^-10^, β = 0.033) and located ~21kb upstream of miR-339-3p (**Figure 3B**), and rs7607369 associated with BMI (p = 1.10×10^-7^, β = -0.016) and located ~11.7kb upstream of miR-26b-5p (**Figure 3C**). These three SNPs were previously identified as miR-eQTLs that change the expression levels of related miRNAs in blood (Huan et al., 2015). In addition, rs4722551 located ~2kb upstream of miR-148a shows the strongest association with LDL (p = 3.95×10^-14^, β = 0.039) on the Chr7p15.2 locus (**Figure 3D**).

Moreover, rs174561 has previously been reported by (Nikpay et al., 2019) to change the expression of miR-1908-5p. We found this SNP, located in the coding sequence of miR-1908-5p, to be associated with lipid traits (LDL, HDL, TC, and TG), and rs11614913, located in the coding sequence of miR-196a2-3p, to be associated with WHR. These two variants have previously been reported to be associated with lipid traits and WHR and have been suggested to change the miRNA structure and expression (Ghanbari et al., 2015). We also found a suggestive association between rs58834075, located in the pre-miR-656 sequence (T > C, Chr14:101066756) and T2D (p = 6.30×10^-5^, β = -0.170). The miRNA secondary structure and free energy changes of both wild-type and mutant alleles of these three SNPs (rs174561, rs11614913 and rs58834075) are illustrated in **Supplementary Figure S1**.

### Identification of Methylation Quantitative Trait Loci (me-QTLs)

We identified 29 *cis*-me-QTL effects for 47 independent CpGs at FDR < 0.05 (49 SNP-CpG pairs). Among these, we found 14 *cis*-me-QTLs that were associated with both the expression level of 8 miRNAs and the methylation level of 26 CpGs (**Supplementary Table S5**). In total there were 7 *cis*-me-QTLs (for 8 CpGs) that were associated with a cardiometabolic trait in the current study (**Table 3**). Furthermore, 4 *trans*-me-QTL effects for 21 independent CpGs were found at FDR < 0.05 (27 SNP-CpG pairs) (**Supplementary Table S5**). Two out of the four *trans*-me-QTL were miR-eQTL SNPs (rs174548 for miR-1908-5p and rs1997243 for miR-339-3p). None of the associated CpGs *in trans* were found in the current study to be associated with cardiometabolic traits.

**Table 3:** Identified me-QTLs with cardiometabolic-associated CpGs.

miRNA	SNPID	CpG	Cis^†^/Trans	miR-eQTL SNP^*^	SNP associated with cardiometabolic trait	CpG associated with cardiometabolic trait
**miR-611**	rs174538	cg16150798	Cis	–	FG, LDL, HDL, TG, TC	WC
**miR-588**	rs9388486	cg20229609	Cis	–	T2D	SBP, DBP
**miR-1908-5p**	rs174548	cg03921599	Cis	√	FG, HbA1c, LDL, HDL, TG, TC	LDL, TC
**miR-199a-1**	rs3786719	cg02907064	Cis	–	LDL, TC	LDL
**miR-6745**	rs901750	cg00724111	Cis	–	HDL	FI, SBP, DBP
**miR-8073**	rs3809346	cg22382805	Cis	–	CAD	FI
**miR-653, miR-489**	rs2528521	cg06934092	Cis	–	BMI	FG, FI, TC
**miR-8073**	rs3809346	cg19700260	Cis	–	CAD	DBP

Shown are 7 me-QTLs associated with methylation levels of 8 cardiometabolic-associated CpG sites.

*miR-eQTL SNP is associated to change the expression of miRNA level.

^†^SNP and CpG are located not further away than 250kb.

### Testing DNA Methylation and Expression of miRNAs Associated With Cardiometabolic Traits

To access the relationship between miRNAs and cardiometabolic traits in other omics layers, we performed a candidate-based test to check whether the 67 identified miRNAs, with SNPs associated with cardiometabolic traits, show also association between DNA methylation and miRNA expression with cardiometabolic traits. Using DNA methylation data from 1,438 RS participants, we found 278 CpG sites annotated to 64 out of the 67 miRNAs, to be associated with any cardiometabolic trait (**Supplementary Table S6**). By integrating our DNA methylation results with the GWAS data, we observed an overlap of 38 miRNAs (79 CpGs) that had both a SNP and a CpG associated with the same trait (**Supplementary Table S7**). The CpG site showing the most significant association was cg15616915 which is located in the regulatory region of miR-26b and is positively associated with TG (p = 1.59×10^-4^, β = 0.009). We found 16 cardiometabolic-associated CpGs that are annotated to more than one miRNA. For example, cg03722243 associated with BMI (p = 1.55×10^-3^, β = 0.001) is annotated to miR-489 and miR-653, which are clustered on chromosome 7. In addition, cg15334028 associated with WC, HDL, LDL, and TG is annotated to three miRNAs (miR-638, miR-6793, and miR-4748) on chromosome 19.

We identified two CpGs that are associated with the expression level of miRNAs (miR-eQTM) at FDR < 0.01. The most significant cis-miR-eQTM, cg26363555 has been reported to be negatively associated with both miR-125b-5p (~2kb downstream) and miR-100-5p (~50kb upstream) expression levels (Huan et al., 2019). The CpG cg26363555 was positively associated with FG (β = 0.012) and DBP (β = 2.00x10-4) and negatively associated with HDL (β = -0.004) in the RS. In addition, cg03891346 has been reported to be negatively associated with the expression level of miR-100-5p (~53kb downstream) (Huan et al., 2019). This CpG, which is also annotated to MIR125B1, was positively associated with WC (β = 5.00x10^-4^) in the RS.

**Figure 4:**
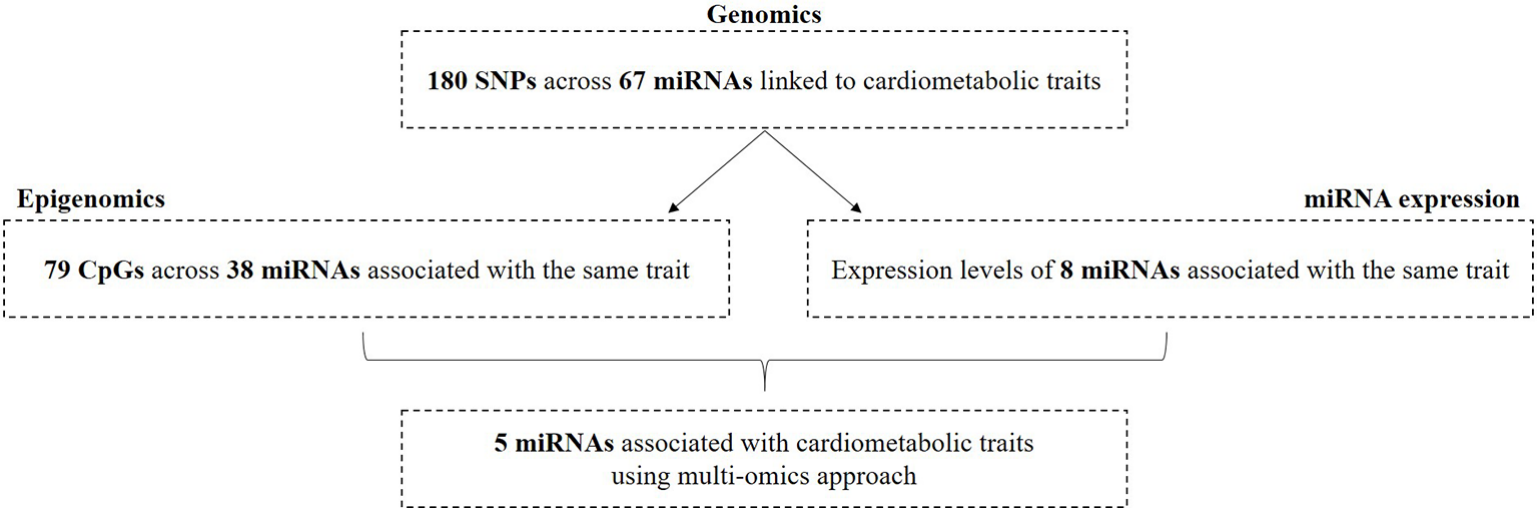
Overview of miRNAs associated with cardiometabolic traits by integrating three omics layers.

Next, we tested whether the 67 identified miRNAs show differential expression in plasma in relation to the associated cardiometabolic trait(s). Of the 67 miRNAs, we could only test the association of 28 mature miRNAs that were well-expressed in plasma and of which the phenotype of interest was available in the RS. Of these, plasma levels of 22 miRNAs were nominally associated with at least one cardiometabolic traits (**Supplementary Table S8**). Furthermore, out of the 67 miRNAs, we found 12 differently expressed mature miRNAs to be associated with the same trait (**Table 4**). Plasma levels of miR-126-3p, miR-126-5p, miR-10b-5p, miR-148a-3p, miR-199a-1-3p, miR-199a-1-5p, miR-125b-5p, and miR-100-5p were positively associated with serum TC levels. In contrast, miR-6886 was negatively associated with serum TC levels. A negative association between miR-126-5p and miR-126-3p and CHD was found. Furthermore, we observed a negative association between miR-4681 levels and WC. An overview of the number of associated miRNAs using different omics data is illustrated in **Figure 4**.

**Table 4:** Plasma expression levels of miRNAs associated with cardiometabolic traits.

miRNA	β-coefficient	P value	Associated trait
**miR-126-3p**	0.379	1.09x10^-14^	TC^†^
**miR-10b-5p**	0.352	3.30x10^-11^	TC^†^
**miR-126-5p**	0.258	3.75x10^-11^	TC^†^
**miR-148a-3p**	0.189	8.01x10^-06^	TC^†^
**miR-199a-1-3p**	0.171	3.38x10^-05^	TC^†^
**miR-125b-5p**	0.159	2.43x10^-03^	TC^†^
**miR-100-5p**	0.141	3.15x10^-03^	TC^†^
**miR-6886-3p**	-0.083	9.49x10^-03^	TC^†^
**miR-126-5p**	-0.365	1.24x10^-02^	CHD^‡^
**miR-4681**	-0.440	2.13x10^-02^	WC^*^
**miR-199a-1-5p**	0.074	3.38x10^-02^	TC^†^
**miR-126-3p**	-0.385	3.54x10^-02^	CHD^‡^

Model 1: adjusted for: age, gender, current smoking.

*Model 1 + BMI.

^†^Model 1 + BMI, lipid medication.

^‡^Model 1 + BMI, blood pressure lowering medication, lipid medication.

Furthermore, out of 22 miRNAs that were associated with at least one cardiometabolic trait, we found validated target genes for 14 miRNAs. We tested the association between these target genes and cardiometabolic traits using summary statistics GWAS data. After correcting for multiple testing, based on the number of tested SNPs in the target genes of a miRNA, we found 24 unique target genes for 9 of the 14 miRNAs to be associated with cardiometabolic traits (**Supplementary Table S9**).

Finally, we sought overlapping miRNAs that were associated with the same cardiometabolic trait in the three different omics analyses (**Supplementary Table S10**). Since not all related phenotypes were available within the RS and not all miRNAs were expressed, we tested 64 miRNAs that had DNA methylation sites and 22 mature miRNAs that were available for miRNA expression analyses using the RS. We found five miRNAs, including miR-10b-5p, miR-148a-3p, miR-100-5p, miR-125b-5p, and miR-6886 that had at least one CpG and of which the expression was also associated with the same cardiometabolic trait. After prioritization based on the suggested criteria for potential functionality, miR-10b-5p, miR-148a-3p, miR-125b-5p, and miR-100-5p were highlighted as the most likely miRNAs involved in the pathogenesis of risk factors for T2D and CHD (**Table 5**).

**Table 5:** Cardiometabolic-associated miRNAs after integrative multi-omics data and in silico prioritization analysis.

miRNA	Associated traits	SNPID	P value	Proxy SNPs (Non-syn.)	GWAS	miRNA CpG	P value	Associated trait	miR-eQTM	Associated trait	miRNA Exp.	Associated trait
miR-10b-5p*	LDL^†^, TC, CAD, T2D	rs532436(‡)	4.02x10^-30^	9 (0)	√	cg25820279	4.37x10^-02^	TC	–	–	3.30x10^-11^	TC
miR-148a-3p*	LDL^†^, TC, TG	rs4722551	3.95x10^-14^	1 (0)	√	cg18188200	1.94x10^-03^	TC	–	–	8.01x10^-06^	TC
miR-125b-5p*	TC^†^, LDL, HDL	rs7117842(‡)	2.48x10^-15^	52 (0)	√	cg06749053	2.39x10^-02^	LDL	cg26363555	HDL	2.43x10^-03^	TC
miR-100-5p*	TC^†^, LDL, HDL	rs7117842(‡)	2.48x10^-15^	52 (0)	√	cg14724899	4.02x10^-02^	HDL	–	–	3.15x10^-03^	TC
**miR-6886-3p**	LDL^†^, TC, CAD	rs17248720	2.40x10^-148^	49 (0)	–	cg19751789	8.03x10^-04^	TC	–	–	9.49x10^-03^	TC

*MiRNAs which were associated with cardiometabolic traits using multi-omics approach and were according to the prioritization analysis more likely to be involved in the development of cardiometabolic traits. ^†^Trait to which the SNP is associated. ^‡^miR-eQTL SNP.

Shown here are 5 miRNAs that were association with cardiometabolic traits using multi-omics approach and being tested with additional in silico analysis. Proxy: Number of proxy SNPs (R² > 0.8) in strong linkage disequilibrium (LD) with the given variant. Non-synonymous: Number of non-synonymous variants that are in high LD with the given variant. GWAS: (√ SNP represented to be top SNP with the strongest association with the related trait on the certain genomic position). miR-eQTM: CpG is associated with expression level of miRNA.

## Discussion

In this study, we integrated different population-based omics data (including genetics, epigenetics and miRNA expression) to identify miRNAs associated with cardiometabolic traits. Genetic variants related to 67 miRNAs were associated with the studied traits. Alterations in DNA methylation of CpG sites annotated to 38 of these miRNAs and plasma expression levels of 8 of them were also associated with the same trait. In principle, the association between a miRNA and trait of interest in more than two layers of omics may strengthen its potential to play a role in the disease underlying mechanisms. In this context, we sought to identify overlap between miRNAs that were associated with the same cardiometabolic trait across different approaches. This integration analysis revealed the correlation between four miRNAs (miR-10b-5p, miR-148a-3p, miR-125b-5p, and miR-100-5p) and lipid traits.

MiR-10b-5p is a highly conserved miRNA across multiple species and is located inside the homeobox D cluster on chromosome 2. A recent study showed a mediating role for miR-10b between obesity and primary breast cancer (Meerson et al., 2019). Moreover, previous research in mice found a negative regulatory role of miR-10b on cholesterol efflux *via* targeting the ATP binding cassette transporter gene (ABCA1) (Wang et al., 2012). MiR-10b has been also shown to be involved in the progression of atherosclerosis, which is a major cause of cardiovascular disease (Wang D. et al., 2018). We found a genetic variant (rs532436;A > G) annotated to the Alpha 1-3-N-acetylgalactosaminyltransferase (*ABO*) gene to be positively associated with LDL, TC, CAD, and T2D. The *ABO* gene has been linked to cholesterol absorption and cardiovascular disease (Silbernagel et al., 2013). Rs532436, located on chromosome 9, has been reported as *trans*-miR-eQTL for miR-10b-5p (Nikpay et al., 2019). In this study, we further showed that a CpG site (cg25820279) annotated to Homeobox D3 (*HOXD3*), is located in the regulatory region of miR-10b and is associated with total cholesterol levels in serum. In addition, the expression level of miR-10b-5p in plasma showed a positive association with total cholesterol levels, which further support the crucial role of miR-10b-5p in lipid metabolism.

MiR-148a-3p has been shown to control the LDL uptake and cholesterol efflux through affecting the expression of low-density lipoprotein receptor (LDLR) (Goedeke et al., 2015). Moreover, *in vivo* studies in mouse models have confirmed that miR-148a-3p is upregulated in adipogenesis and highly expressed in liver tissue (Gailhouste et al., 2013). We found rs4722551, located ~2kb upstream of miR-148a, associated with LDL, TC and TG. It has been suggested previously that a large part of regulatory elements such as promoter regions are located within +/-2kb of pre-miRNAs (Saini et al., 2007). Rs4722551 has previously been reported to be positively associated with serum lipid levels *via cis*-miR-eQTL in liver tissue (Wagschal et al., 2015). Our findings may shed light on the mechanism that associates the rs4722551 risk allele (T > C) with an increased miR-148a-3p expression, which is subsequently associated with higher serum cholesterol levels. Furthermore, our results showed a CpG site (cg18188200) in the regulatory region of miR-148a to be associated with LDL, TC, and TG and demonstrated that the plasma expression level of miR-148a-3p is also associated with total serum cholesterol levels. These data are in line with the findings from previous studies reporting a functional role for miR-148a-3p in lipid metabolism confirmed by various *in vivo* and *in vitro* validation experiments (Goedeke et al., 2015; Wagschal et al., 2015).

We found strong associations of rs7117842, located ~512kb upstream of miR-100-5p/125b-5p, with TC, LDL, and HDL, suggesting these two miRNAs to play a role in lipid metabolism. The SNP has been previously shown to be negatively associated with the expression levels of miR-100-5p and miR-125b-5p in blood (Huan et al., 2015). In our analysis, plasma expression levels of miR-100-5p and miR-125b-5p are positively associated with TC. These findings could be interpreted in a way that carrying the risk allele of rs7117842 (T > C) is associated with decreased expression of miR-100-5p/125b-5p, which is associated with a reduced increase of total serum cholesterol levels. In addition, cg26363555, located in the promoter region of miR-125b-5p, was previously reported to act as miR-eQTM by changing the expression levels of both miR-100-5p and miR-125b-5p (Huan et al., 2019). We found cg26363555 associated with HDL in the RS. In addition, cg03891346 annotated to MIR125B1 was reported to be associated with the expression level of miR-100-5p (Huan et al., 2019). Our DNA methylation analysis results showed the association between cg03891346 and waist circumference in the RS. Our findings are partly in line with previous research investigating the role of miR-125b-5p on adipogenesis where it is observed that miR-125b-5p downregulates the anti-adipogenic gene *MMP11* in human, indicating that miR-125b-5p *via MMP11* positively regulate adipogenesis (Rockstroh et al., 2016). Conversely, the same study demonstrated a direct effect of reduction in fat accumulation through overexpression of miR-125b-5p (Rockstroh et al., 2016). In addition to the role of miR-125b-5p on lipid metabolism in human, its regulatory role has been investigated in other organisms including zebrafish and mice. Over-expression of miR-125b in zebrafish is linked to lipid metabolism in brain, heart and liver tissue (Wang X. et al., 2018). This study observed that overexpression of miR-125b inhibits osteoblastic differentiation and promotes fat synthesis. Moreover, the expression of miR-125b is activated by estrogen *via* ERα *in vitro* and *in vivo* in mice, in which they demonstrated that miR-125b can limit fat accumulation in liver tissue (Zhang et al., 2015). These contradictory findings may implicate that miR-125b-5p plays an important role in lipid metabolism *via* a complex molecular cascade. However, the role of miR-100-5p in regard to lipid metabolism and cardiovascular disease yet to be further investigated. Since miR-100-5p and miR-125b-5p are located in the same locus on chromosome 11, it could be possible that miR-125b-5p is the driving miRNA in relation to the associated lipid traits. Future research is warranted to confirm the regulatory role of miR-100-5p in lipid metabolism.

The main strengths of this study include the use of robust data from the large-scale GWAS studies and multi-omics implementation of a large sample size in the Rotterdam Study, which indicates with more confidence that miRNAs are involved in the pathophysiology of cardiometabolic diseases. Our study, however, does not come without limitations. First, our study design is based on associations rather than causations, therefore this approach does not prove that the identified miRNAs play a causal role in the studied traits. To test for causal inferences between miRNAs and disease risk, future studies should test mediating effects and incorporate functional follow-up experiments. Furthermore, our study design was based on a cross-sectional approach, which means that individuals included in this study were not free of CHD or T2D. In regard to test whether the identified miRNAs are associated with the risk of developing disease, future longitudinal studies are warranted. Another limited factor is that we were unable to link all identified miRNAs with epigenetic and expression analyses in the RS, since not all phenotypic data were available for each trait of interest nor were all miRNAs well-expressed in plasma. In addition, different sub cohorts of the RS were used for DNA methylation and miRNA expression analysis due to the availability of data. DNA methylation and miRNA signatures are dynamic over time and could have yield in confounding results. The challenge of this multi-omics approach includes the intra-individual variation and thereby lack of generalizability between datasets. However, the sub cohorts of RS-II and RS-III are extensions of the RS-I cohort. Previous epigenetic (DNA methylation) studies using the RS data showed that the results are replicated after additional adjustment for sub cohort (Ligthart et al., 2016; Braun et al., 2017; Nano et al., 2017). This may indicate that the intra-individual differences between variables in these RS sub cohorts have not significantly affected by exposing to different environmental factors. Yet in an optimal setting one should apply the multi-omics analysis in the same individuals and the same timeframe. Furthermore, we used whole blood to determine DNA methylation and plasma to check expression levels of miRNAs, which are not the most relevant tissue for cardiometabolic traits. This could have resulted in overlooking some of the miRNAs, but the found associations are comparable because both analyses were performed in the same tissue. In an optimal setting one should examine the observed associations using next-generation sequencing covering all miRNAs in target tissues (e.g., adipose tissue, heart, pancreas and liver). Such infrastructure is not yet available in large epidemiologic studies with validated clinical data. However, for the use of miRNAs as targets for early diagnosis or progression of T2D and CHD, blood might be a very good test tissue since it is a non-invasive method for biomarker measurements in clinical diagnosis. In addition, regarding potential missed cardiometabolic-associated SNPs, our study could have benefited from denser genotyping methods including 1000 Genomes project or the Haplotype Reference Consortium (HRC).

## Conclusion

In this study, we systematically examined the association of miRNAs with cardiometabolic risk factors and diseases using population-based genetic, DNA methylation and miRNA expression data. By integrating these omics data we found several cardiometabolic- associated miRNAs, such as miR-10b-5p, miR-148a-3p, miR-125b-5p, and miR-100-5p involved in lipid metabolism, that can be viewed as potential biomarkers for early diagnosis or progression of T2D and CHD. Future experimental studies are warranted to elucidate pathways underlying the link between these miRNAs and cardiometabolic risk factors such as dyslipidemia, central adiposity and elevated blood glucose levels.

## Data Availability Statement

All datasets analyzed for this study are included in the article/**Supplementary Material**.

## Ethics Statement

The Rotterdam Study has been approved by the institutional review board (Medical Ethics Committee) of the Erasmus Medical Center and by the review board of The Netherlands Ministry of Health, Welfare and Sports. The patients/participants provided their written informed consent to participate in this study.

## Author Contributions

MG designed the study. MM and SM performed analysis. MM, SM, and MG wrote manuscript. JK, GW, PK, JB, JM, AU, MI, and MK provided resources and data. All authors contributed to revising and finalizing the manuscript.

## Funding

The Rotterdam Study is funded by the Erasmus Medical Center and Erasmus University, Rotterdam, the Netherlands Organization for the Health Research and Development (ZonMw), the Research Institute for Diseases in the Elderly (RIDE), the Ministry of Education, Culture and Science, the Ministry for Health, Welfare and Sports, the European Commission (DG XII), and the Municipality of Rotterdam. The DNA methylation data was funded by the Genetic Laboratory of the Department of Internal Medicine, Erasmus MC, and by the Netherlands Organization for Scientific Research (NWO; project number 184021007). MiRNA expression analyses by HTG EdgeSeq WTA was funded by Johnson & Johnson.

## Conflict of Interest

JK, GW, PK and JB are employees of Johnson & Johnson. The Johnson & Johnson company supported the microRNA data collection.

The remaining authors declare that the research was conducted in the absence of any commercial or financial relationships that could be construed as a potential conflict of interest.
