# Supplementary material for: Multi-Omics Analysis Reveals MicroRNAs Associated With Cardiometabolic Traits
Source: Front Genet. 2020 Feb 27;11:110. doi: 10.3389/fgene.2020.00110 (PMC7056871; doi:10.3389/fgene.2020.00110)
Supplement: Supplementary file 1 [file DataSheet_1.pdf]

Supplementary Material

1 Supplementary Data

Measurements on cardiometabolic risk factors and diseases in the Rotterdam Study

Anthropometric measurements: Participants were measured in standing position in light clothes without shoes. Waist circumference was measured midway between the low rib margin and iliac crest. Measurements of hip circumference were measured at the point resulting in the maximum circumference over the buttocks. Body-mass index was calculated as weight (kg) dived by the square of height (m). Waist-hip ratio was calculated as waist circumference divided by hip circumference.

Type 2 Diabetes and glycemic traits: Type 2 diabetes was defined according to the current WHO guidelines: fasting blood glucose levels ≥ 7.0 mmol/ L, non-fasting blood glucose level of ≥ 11.1 mmol/L (when fasting samples were unavailable), or the use of blood glucose lowering medication. Information about medication was obtained from both questionnaires during home visits and pharmacy dispensing records. Concentrations of fasting glucose, non-fasting glucose, and fasting insulin levels were measured in serum (mmol/L) from blood samples obtained at the visit to the research center. Parameters were measured with a Roche Modular P800 at Erasmus MC AKC laboratory.

Lipid traits: Blood samples of participants were obtained during the visit to the research center. Using automatic enzymatic method levels of high-density lipoprotein (HDL), total cholesterol (TC) and triglyceride (TG) were measured in serum (mmol/L). Low-density lipoprotein (LDL) concentration was calculated via the Friedewald formula (TC-HDL-(TG/5)). Information about medication was obtained from both questionnaires during home visits and pharmacy dispensing records.

Cardiovascular traits: Systolic and diastolic blood pressure was measured (mmHg) in a sitting position after a period of rest and the mean value was taken as a phenotype for this analysis. Prevalent coronary heart disease was defined as myocardial infarction, coronary artery bypass graft surgery or percutaneous coronary intervention. Information about medication was obtained from both questionnaires during home visits and pharmacy dispensing record.

2 Supplementary Figures

Supplementary Figure I. Predicted secondary structure of miRNA wild type and variant. Location of the SNP is demonstrated by an arrow. The red part shows the mature sequence and the blue part shows the rest of the pre-miR. The corresponding minimum free energy (MFE) is illustrated with the thermodynamic ensemble ΔG. **A**, Secondary structure of miR- 196a2-3p wildtype and variant (rs11614913) located in mature miRNA sequence. MFE changes by -4.6kcal/mol. **B,** Secondary structure of miR-656 wildtype and variant (rs58834075) located in precursor miRNA sequence. MFE changes by -0.6kcal/mol. **C,** Secondary structure of miR-1908-5p wildtype and variant (rs174561) located in precursor miRNA sequence. MFE changes by +3.2kcal/mol.

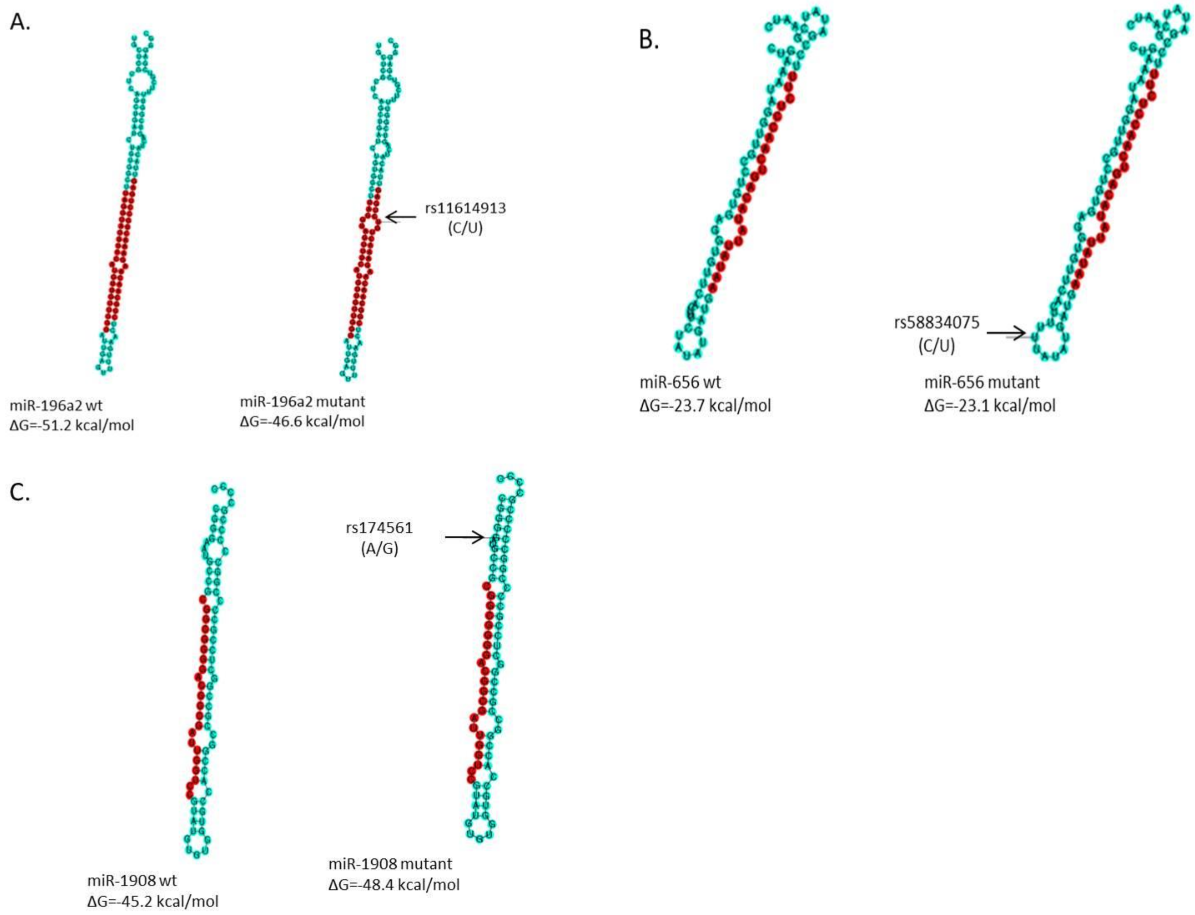

3 Supplementary Tables

Supplementary Table 1. Description of GWAS meta-analysis and consortia used in this study

**Phenotype**	**Consortia**	**Sample size**	**All candidate SNPs in GWAS**
Anthropometric traits			
Body-mass index	GIANT	241,258	2,549
Waist to hip ratio	GIANT	241,258	2,531
Waist circumference	GIANT	241,258	2,533
Glycemic traits			
Glucose fasting	MAGIC	133,010	2,639
Glucose after 2h	MAGIC	45,854	48
Insulin fasting	MAGIC	108,557	2,637
Pro-insulin	MAGIC	10,701	2,506
HbA1c	MAGIC	123,665	887
HOMA-IR	MAGIC	46,186	64
HOMA-β	MAGIC	46,186	37
Type 2 diabetes	DIAGRAM	26,676 cases/ 132,532 controls	10,690
Lipid traits			
Low-density lipoprotein	GLGC	173,000	2,377
High-density lipoprotein	GLGC	187,000	2,385
Total cholesterol	GLGC	187,000	2,385
Triglyceride	GLGC	178,000	2,376
Cardiovascular traits			
Coronary artery disease	CARDIoGRMplusC4D	60,801 cases/ 123,504 controls	10,706
Diastolic blood pressure	ICBP	71,255	2,345
Systolic blood pressure	ICBP	71,255	2,345

Supplementary Table 2. Participant characteristics of the Rotterdam Study for DNA methylation analysis and miRNA expression profiling

	DNA methylation (RS-II-3 & RS-III-2)	DNA methylation (RS-III-1)	miRNA expression (RS-I-4 & RS-II-2)	P value*
N	717	721	1999	
Female	413 (57.6%)	391 (54.2%)	1141 (57.1%)	<0.001
Age (years)	67.5 (5.93)	59.8 (8.16)	71.6 (7.58)	<0.001
BMI (kg/m^2)	27.7 (4.12)	27.6 (4.63)	27.7 (4.11)	0.905
Waist circumference	94.4 (12.00)	93.75 (12.92)	93.6 (11.98)	0.142
WHR	0.9 (0.09)	0.9 (0.08)	0.9 (0.09)	<0.001
Current smoking (yes)	76 (10.6%)	193 (26.8%)	288 (14.4%)	<0.001
Triglycerides (mmol/L)	1.5 (0.79)	1.5 (0.88)	NA	0.298
HDL-cholesterol (mmol/L)	1.5 (0.44)	1.4 (0.41)	1.4 (0.39)	<0.001
LDL-cholesterol (mmol/L)	3.7 (0.94)	3.9 (1.00)	NA	<0.001
Total cholesterol (mmol/L)	5.5 (1.02)	5.6 (1.07)	5.6 (0.99)	0.004
Lipid lowering medication (yes)	225 (31.4%)	191 (26.5%)	450 (22.5%)	<0.001
Systolic blood pressure	144.8 (21.91)	134.2 (19.76)	148.2 (20.82)	<0.001
Diastolic blood pressure	84.4 (11.66)	82.8 (11.38)	79.6 (10.84)	<0.001
Coronary heart disease	28 (3.9%)	46 (6.4%)	214 (10.7%)	<0.001
Anti-hypertensive medication (yes)	310 (43.2%)	217 (30.1%)	880 (44.0%)	<0.001
Glucose (mmol/L)	5.7 (1.11)	5.6 (1.04)	5.8 (1.09)	0.001
Insulin (pmol/L)	82.6 (48.26)	96.0 (63.04)	NA	<0.001
Prevalence type 2 diabetes	96 (13.4%)	74 (10.3%)	278 (13.9%)	0.04
Anti-diabetic medication	59 (8.2%)	39 (5.4%)	132 (6.6%)	0.0985

Values are presented as mean ±(SD) or N (%).
*Differences between groups were addressed using ANOVA in the case variables were available among three groups, Student's T-tests in the case variables were available in two groups.
NA: Not Available

frontiers
